# Commentary on the Hindu System of Medicine

**Published:** 1846-10-01

**Authors:** 


Commentary on the Hindu System of Medicine. By *T. A. Wise*, M.D. &c., Bengal Medical Service. 8vo. pp. 431. Calcutta, 1845.

From an early period of Dr. Wise's residence in Bengal, he has been in the habit of employing his leisure time in studying the history of medical science in different ages and nations of the earth. His attention was naturally directed, in an especial manner, to the examination of the Hindu Shastras or ancient records of medicine in the East. The present volume is the result of his researches upon this subject. It is only of late years that the European student has been even so much as aware that there were any writings, at all, on medicine among the Hindoos. Sir William Jones, the celebrated Oriental scholar, had asserted "that there is no evidence that, in any language of Asia, there exists one original treatise on medicine as a science;" and Mr. Mills, the learned historian of India, has made a similar statement. That such a belief is, however, far from being correct, has been abundantly shewn by the recent writings of Professor Wilson, and of Drs. Heyne, Ainslie and Boyle; and now the work of our author presents us with an abridgement of an almost entire system of medical science, if science it may be called, extracted from the Shastras.

While the greater part of Europe was in a state of savage ignorance, there is unquestionable evidence to prove that the inhabitants of Hindostan and of the surrounding countries had attained to high civilization, and had made great progress in most of the arts and sciences of life. Medicine was one of these, which seem to have been studied with much care and diligence. The most ancient medical work, which is extant (partially so, at least), is called the Ayur Veda, and is said to have been given by Brahma himself to the world, in compassion to man's ignorance and suffering. This sacred work contains, among other subjects, a description of the structure of the human body derived from anatomical examination; for there is abundant evidence to show that dissection of the human body was practised in these early times.

It is unnecessary for us even to give the names of the other medical books, as the information cannot be of interest to the majority of our readers; those, who wish to know particulars, cannot have recourse to a better source than our author's work.

The medical caste, *par excellence*, among the Hindoos is known by the name of Vaidhyas, *i. e.* those who understand the "Ayur-ved" or medical shastre. The Vaidhyas are the proper teachers or professors of the art. Many of the precepts, which they inculcate, would do no discredit to our most learned doctors. For example, the importance to the physician of combining practical experience with attentive study of books is thus very emphatically enjoined:—

"Without a knowledge of books he will be confused, like a soldier afraid in the time of action, will be a great sinner, and should be capitally punished by the rajah. On the other hand, a want of practical knowledge will impede its advancement, and his senses will be bewildered, when called on to treat acute diseases. Such a physician will not be esteemed by the great, as he cannot practice with success when only instructed in half his duty. Such a person is the murderer of his species, and the medicine prescribed by him may be compared to poison, or lightning—such ignorance prevents all the good effects of remedies. As the two wheels of a chariot, or the two wings of a bird, assist in their progress, so will the knowledge of the shastres, and of practice, lead the physician to proceed with safety and success in the treatment of the diseased; but, should the physician want either of these essential qualifications, his progress will be impeded, as one wing or one wheel will impede the progress of the bird, or the chariot. It is the combination of both these qualifications which is required; when medicine becomes like the water of immortality (*Amrita*). Such a physician, if he is to acquire celebrity, must still daily endeavour to improve his mind by an attentive perusal of scientific books." P. 18.

Again, is there not sound advice, alike to patient and doctor, in the following remarks?

"Some severe diseases are cured immediately, by a good physician; but simple diseases are increased much by the want of early assistance. At the commencement, like a young plant, it is readily rooted up; but, as it expands and grows in strength, the difficulties are much increased. Even for a slight disease, the assistance of a practitioner will be of much use; for as a large man at the bottom of a pit may get out by long-continued exertion, his extrication will be much facilitated by the assistance of a friendly hand. As in war, a sword may defend many, so in the hand of an enemy it will destroy. In like manner, the shastres and water may become the cause of destruction instead of benefit to mankind." P. 19.

*Mercury*, we suppose, is regarded as the greatest of all remedies; for we are told that the physician, who knows the value of this metal, is like a god; he, who knows the qualities of herbs and roots, is like a man; he, who knows the use of knife and of fire, resembles a demon; and he, who knows the proper prayers to be offered up in the time of sickness, is like a prophet. A bad or ignorant physician may indeed sometimes cure a patient, but he does not consider the thousands he has killed ; such a one is like a boat in a storm without a pilot, or a blind man in the performance of any work, and is to be looked on as an angel of death.

Some of the duties inculcated upon a physician are certainly rather strange. For example, he is directed never to look at the rising or the setting sun ; he should not eat or drink out of a broken vessel, nor sleep with his face to the earth! As a matter of course, too, great respect is to be paid to various omens. Thus, we read that, "before a physician visits a patient, he should first remark the position he is in when the messenger arrives to consult with him; and, by the person's countenance and conversation, endeavour to ascertain whether or not the patient will survive. As he proceeds to visit the sick person, he must carefully note any good or bad omens that may occur regarding the messenger, the flight of birds, the relative position of animals, &c. Seeing cows, or Brahmans on the right-hand side are favourable; as also corpses, jackals, vessels of water, &c., when seen on the left side. It is unfavourable when lizards are heard upon leaving the house, when vultures or bad characters are seen, or when the Physician is called by another person, or is hit by anything behind, or when a person sneezes."

In the examination of patients, most minute attention is directed to be paid to the state of the various excretions, among other signs or indications of disease. "Unless the disease," it is sagaciously remarked, "is well explained, seen and known, the practitioner will not understand it, and will be made foolish by his ignorance ; whereas the knowledge and judgment of the physician, like a lamp which illuminates a room, enable him to understand the nature of the disease of the body."

There is an odd mixture of truth and error, of sound morality and wicked folly, in the following description of the *Recompence of the Physician.*

"When a physician has cured a disease, he is entitled to the usual gifts for the performance of a good action. These will vary with the rank and condition of the patient. Money will be the recompence bestowed by the rich ; friendship, reputation, increase of virtue, prayers, and gratitude will be that of the poor. When a Guru, a Brahman, or a Dandi, a relative, a humble and good friend, or one without relations consults a physician, he must not accept of any pecuniary recompence: His reward in such cases will be an incresse of knowledge, and the gratification of his desires in having an opportunity of performing a good action. His cures will ensure the admiration, and the esteem of all men; he will be honoured and respected as a master, and after death he will go to heaven. Should the patient prove ungrateful after being cured, his holyness and good fortune will pass to the physician. But the physician must avoid administering remedies, to hunters or great sinners. Such people do not deserve his assistance." P. 29.

This and the preceding extracts are taken from Book I, on the History of Medicine, the character and duties of physicians and their pupils, &c.

The Second Book professes to treat of Anatomy and Physiology, and to describe the elements of the body; its generation and growth ; the structure of its various parts; its spiritual elements; as well as the different temperaments, death, &c.

The influence of *longing* on pregnant women is very particularly insisted upon:

" During Pregnancy, if the woman is not gratified with what she wishes to eat, and the air is deranged, the child will be crooked and cripple, or will be small in size, dumb, or cannot speak distinctly; will be blind, or have his eyes defective, or will be an unbeliever in the sacred shastres. In other such cases these defects are produced by acts of previous wickedness of his own, or of his parents in a former state of existence.

" Whatever is wanted by the pregnant woman should therefore be supplied, when a perfect child will be born. If the woman desires to see a Rajah, the child will be great and rich; should the mother wish to adorn herself, the child will be well formed and vain; should the mother wish to see a holy man, the child will be holy and just, and if she longs to see ferocious animals, the offspring will be of that description. In like manner, the desire for particular sorts of food indicates the disposition of the infant, and the form of his body. When the mother wishes to eat buffalo's flesh the child will have blood-shot eyes, much hair, and he will be warlike; and when hog's flesh, he will be sluggish, and sleepy." P. 35.

The Chapter on Death contains some really fine and very just reflections:

" Death is always near; and when it occurs, nothing but the sins and virtuous actions, which have been performed, accompany the soul.* ' When a person leaves his corpse, like a log or a lump of clay, on the ground, his kindred retire with averted faces; but his virtue accompanies his soul. Continually, therefore, let him collect virtue, for the sake of securing an inseparable companion with which he may traverse a gloom, how hard to be traversed! For, in his passage to the next world, neither his father nor his mother, nor his wife, nor his son, nor his kinsmen, will remain in his company: his virtue alone will adhere to him. Single is each man born; single he dies; single he receives the reward of his good, and single the punishment of his evil deeds.' "† P. 81.

The immortality of the soul is frequently and emphatically dwelt upon; as in the following passage:

" After death, the body is like a house without a tenant; and the five elements slowly separate and join their like; the atoms of earth join the earth, the watery mix with water, &c. Death is therefore called in Sanscrit *Panchatwa*, or separation and passage to the five elements. To promote this separation of the elements after death, which would be defiled if buried, and to purify them in their passage from the body, so as to enable the earth, air, fire, water, and ether, of which the body is composed, to join the mass of the same elements which compose the world, the bodies of Hindus are burnt. ' What then dies? not the body, for it only changes its form; and certainly not the soul! Why then regret the death of relations and friends, if they have passed through life with pro-

* " A mansion infested by age and by sorrow, the seat of maladies, harassed with pains, haunted with the qualities of darkness, and incapable of standing long; such a mansion of the vital soul let its occupier always cheerfully quit." Menu, Ch. vi, § 77, p. 183.

† " Menu Chap. IV, § 239, 240, 241, 243. The same idea is thus expressed in another Sanscrit work. " The wise man meditates on the acquisition of knowledge and riches, as if not subject to sickness or death; and cultivates virtue, as if death had already seized him by the hair." (Hitopadesha).

priety! such grief is indeed natural, for it is universal, but it is the offspring of our ignorance and our selfishness." P. 81.

The Pythagorean doctrine, however, of *metempsychosis* is taught in the subsequent part of this chapter.

The Third Book is devoted to the various subjects that come under the general appellation of Therapeutics, the first department of which is hygienic regimen. All Hindoos anoint their bodies daily with oil: a practice that is considered to be conducive not only to the preservation of health, but also to prosperity and good fortune. It promotes an agreeable perspiration over the whole surface, and guards the system against the injurious effects of vicissitudes of temperature. The Orientals are also much in the habit of using baths of various kinds.

"Independent persons, such as rich merchants, bankers, talukdars and others, generally bathe at 10 or 11 o'clock, and after performing these ceremonies they breakfast. Shop-keepers, day-labourers, &c. eat at 10 o'clock, a handful of rice, which has been moistened for half an hour in water or a handful of gram moistened, and do not generally bathe till after 12, 2, or even 3 o'clock. There are some who bathe twice or thrice a day, but they are few in number. This description applies to the male inhabitants of towns. The higher class of females seldom bathe in rivers, but do so in tanks, in their respective gardens; or in warm water, between 10 and 11 o'clock. Widows of the lower class are not strict, and do not observe the rules of the Shâstras regarding bathing.*" P. 96.

Vapour baths are a favourite remedy for a variety of painful complaints. The patient is first well rubbed with oil, and is then seated over a pot of boiling water, with a covering thrown over both. Sometimes medicinal plants are put into the water.

We cordially say Amen to the soundness of the following remarks upon a most useful, though too often neglected, part of therapeutic treatment:

"The Hindu medical writers usually commence the cure of a disease by arranging the diet that is to be followed by the sick person. So much do the Hindu Physicians rely upon diet that they declare that most diseases may be cured by following carefully dietetic rules;† and if a patient does not attend to his diet, a hundred good medicines will not remove the disease. The generality of diseases being supposed to be produced by derangement of the humours, if one or more are morbidly increased in quantity, their indications of cure are commenced by promoting the just balance of the elements and humours, by a judicious choice of aliments, and by such means as assist the vital principle on the completion of the assimilation. On this account they have not only been careful in describing the regimen, but also the food and drink for the different seasons, and even the vessels in which they should be kept." P. 98.

---

* "The Hindu men and women may be seen proceeding towards the sacred Ganges in the cool of the day, the one with his small copper lota, the other with her antique earthen pot perched upon her head, amidst the rustling of the beautiful palm trees, which almost hide the graceful cupolas of the neighbouring temples. After washing their heads with some of the mud and water of the Ganges, they proceed to clean their teeth with the branch of a tree which they have brought with them. They then wash their bodies, using mud for soap, fill their vessels with water, and return home."

† "Or as Baglivi expresses the same opinion, as the heading of one of his chapters 'de ciborum delectu, sive de methodo curandi morbos quamplures per opportunum ciborum genus, sine ope remediorum.—Op. omnia, T. 11, p. 530."

From the chapter on Surgery, we shall select the description given of the Rhinoplastic operation performed by the Hindoos.

" *When the nose is cut off, or destroyed by diseases.*—The former is a frequent punishment in the native courts. A fresh leaf is first cut of exactly the size of the nose, it is then to be placed upon the cheek, and the necessary quantity of skin and cellular membrane is to be dissected. The nose is then to be scarified, and after dissecting up the flap, it is to be placed upon the raw part of the nose to which it will adhere. Sutures and bandages are applied to keep the parts together. After the bandage has been applied, a couple of wooden canulæ are to be introduced into the nostril to allow breathing, and to support the new nose. A piece of linen cloth previously soaked in oil is to be applied over the bandage. An aperient is then to be given to the patient, and his general health is to be attended to. Should any other deficiency of the nose be present it may be supplied in the usual manner. If the nose should he deformed it may be reduced in size by the knife." P. 189.

Book IV. treats of medical diseases generally and especially. The first subject that we shall notice is that of Small-pox, in order that we may have the opportunity of introducing the following remarks of our author respecting the primary seat or *habitat* of this destructive pestilence.

" It appears that the Chinese as well as the Hindus were familiar with *small-pox* many centuries before the Arabian physicians described it. It was probably conveyed westward by the Persian conquerors of Hindustan; which seems to be a further confirmation of the country from which it originally came, and the manner in which it gradually approached and eventually reached Europe. The distance and the hot deserts through which the only intercourse for so long a period was held, prevented for a time its progress westward; but, as navigation extended, ships from India would frequently touch at the Arabian ports of the Persian Gulph, and Red Sea, where it seems first to have appeared, A.D. 900.

" The description of the small-pox by Rhazes, the distinguished Arabian physician, first drew the attention of the European physicians to the disease.

" Some say it was introduced into Arabia in 572, the year that gave birth to Mohammad; other testimonies seem to accord with the statement that it was at the siege of Mecca (A.D. 569), by Abraham that the Arabians were first affected with the disease.

" The conquests of the followers of Mohammad conveyed the disease to Persia, Syria, and Egypt; and the successful stand made by the inhabitants of Constantinople, for some time prevented the spread of the disease beyond the Hellespont. So completely does this appear to have been the case, that Honus, a resident physician in that city in the tenth century, states that neither the small-pox nor measles were known in his time in Constantinople.

" The whole of the southern coast of the Mediterranean sea had been subdued by the Arabians; but, it was not till the commencement of the eighth century that the disease was introduced into Spain by the Moors. The victorious Saracens overran Spain, crossed the Pyrenese mountains, and inundated the southern provinces of France. They were driven back by Charles Martel; but they left the small-pox and measles with the conquerors. From this source the diseases quickly spread over Europe.

" The Spaniards in their invasion of Hispaniola and Mexico conveyed the same diseases to these countries, where it committed the most extensive ravages. It would thus appear that the small-pox as well as the measles commenced in Asia, and extended to Africa, Europe, and the new World." P. 239.

*Leprosy.*—This loathsome malady is evidently regarded by the Hindoos in nearly the same light in which it was by the Jews of old, as a mark of special Divine chastisement, and as a disease to be cured rather by confession of sins, prayer, and sacrifices, than by any medicinal remedies. "Lepers in this life are supposed to be born again in the next with the disease upon them; and it is believed to be communicable by contact, by breathing the same air, by eating together, by wearing the clothes, or ornaments, of a person labouring under the disease. Whoever speaks disrespectfully or does any other improper action, or commits sins against his *guru* or *bipra* (pandit bráhman); such as, committing adultery with a bráhman's wife, killing a good man, and robbing a person of his estate, will be liable to be afflicted with this disease." P. 259.

Seven varieties of the disease are described in the Hindoo medical writings. The eruption is either in the form of small, white, coppery or red spots, spreading over large surfaces, and covered with a thin mealy dust, (Lepra vulgaris, or L. alphos); or the blotches are livid, resembling a ripe fig, and are usually accompanied with much pain and burning on the surface; or there are patches of tubercles, which are hard and red round their edges, dark in the centre, and attended with severe pain; or the patches are purplish and black, irregular, hard, dry, pricking and painful; or they resemble the seed of the *kunch* (Abrus precatorius), with red and black spots in the centre, and often terminate in suppuration; or they are black, round, and spreading, often accompanied with much itching, burning and pain. It would seem that, under the term Leprosy, a variety of squamous and tubercular diseases, attended with a cachectic state of the system, are comprehended. In the bad forms of the disease, many of the skin-patches become the seat of foul ulcerations, which discharge an offensivs ichorous matter. Sometimes the hands and feet become stiff, immoveable, and drop off with severe pain; the nose falls in; worms breed in the sores; the voice becomes hoarse and unnatural; and the patient at length dies in a state of wretched emaciation and exhaustion. "Leprosy," we are told, "commences first in the skin, and gradually extends deeper and deeper affecting the different essential parts, as flesh, blood, fat, &c. Thus, like the small shoots of the Banian tree, which are at first confined to the surface, they advance deeper and deeper, until they extend over the whole body. In the first stage, when it is superficial, the use of proper diet and medicines may cure it; but when it has extended to a greater distance, the difficulty of curing it becomes much greater."

*Demoniac Possession* is recognised as a distinct disease by Hindoo physicians. It is, on the whole, interesting to read the accounts, however absurd many of the details are, of different kinds of evil spirits that are believed in the East to enter the bodies of men; as we are forcibly reminded, by the perusal, of the analogy between Hindoo belief and many of the statements in Holy Writ, respecting this source of suffering and disease.

The severe form of periodic *Gastrodynia,* described by Dr. Wise, as occurring among the natives of India, appears to us to be of the same nature (though in a much more aggravated degree) as what is not unfrequently met with in this country, in certain kinds of Dyspepsia connected with an atonic condition of the stomach. The pain usually comes on from one to three hours after taking food; it commences with a sense of dull gnawing uneasiness, and goes on increasing until it becomes most distressing and severe. Firm pressure on the epigastric region generally relieves it partially. Eating too, especially solids, mitigates the suffering for a time; and hence the patient is induced—not indeed by the feeling of hunger, but from having found a previous relief in this way—to the very frequently having recourse to food. Persons who have exhausted their strength by excessive mental application, or who have been in the habit of going very long without food, are those who, according to our experience, have generally been the victims of this form of Gastrodynia. The use of warm stomachics and antispasmodics is required at first;* and subsequently chalybeates must be given, in conjunction with bitter infusions. The food should consist chiefly of meat and bread; the drink of brandy and water, or well-brewed porter or bitter ale. Tea must be eschewed; coffee is much better.

There is obviously a good deal of sound rational practice in the following account of the treatment to be pursued in cases of *Intestinal Worms.*

" The treatment is to commence with the administration of an emetic prepared with ghee and *Sarasádi,* which is a mixture formed of various stimulants and bitter remedies. Strong cathartics are then to be given with clysters. The oil of *Biranga,* made of vermifuge seeds, is to be given with salt, and with the decoction of *Biranga.* The patient should at the same time be careful to avoid such food, and other causes which promote the generation of worms. He should take of the juice or paste of the *Pálása* seeds (Butea frondosa), with the decoction of the *Balanga* seeds. The juice of the leaves of *Paribadraka* (Erythrina fulgens), with honey; or the juice of the leaves of *Patura* (Salincha B.) or the powder of *Biranga* with honey. *Biranga* powder should be mixed with the bread and used by the patient. Different preparations of iron, or the powdered root of long-pepper taken with goat's urine are of use. Tin is recommended to be rubbed upon a rough stone, and the small particles thus removed from the mass is taken in whey." P. 350.

On the subject of *Syphilis,* we are informed that, soon after the appearance of the disease in Europe, its ravages extended over a considerable part of Asia and Africa, as well as Europe. In the East it is known by the name, among others, of the " disease of foreigners or strangers." Old Hindoo medical writers have described various maladies of the genital organs; but it is obvious that they were not acquainted with the venereal disease; for there is no word for it in the Sanscrit language. In the modern Sanscrit works, it is called " Faringi Roga," or Portuguese disease. The general belief among the native doctors is that mercury is the only remedy for it. From a variety of facts and considerations, our author comes to the conclusion that Syphilis " was introduced from America to Europe and Asia, as the small-pox and measles seem to have first appeared in Asia, from whence they spread, and committed such ravages in Africa, Europe, and America."

---

* Nothing is usually better than the following mixture:—

℞. Magnesiæ ustæ . . . . . . ʒss.

Aquæ Menth. Piperit . . . ℥v.

Tinct. Rhei Comp. . . . . ʒvj.

——- Hyosciami . . . . . . ʒj.

Æther. Sulphurici . . . . . ʒj. M.

Capt. Cochl. magna omni horâ, urgente dolore.

In taking leave of Dr. Wise, we cannot deprive ourselves of the pleasure of expressing our high admiration of the learning and indefatigable zeal which he has displayed in the composition of the present volume. By many it will be regarded rather as curious than useful; but all have not the same tastes. The medical historian and antiquary will be pleased to have an opportunity of comparing the state of their science in one country and in one age with that in another. We have been reminded, during the perusal of this commentary, of the writings of Paulus Ægineta in not a few respects. Dr. Wise, we are glad to find, promises us another volume, in which he proposes "to trace the decline of the medical profession in India, and the best means of removing the state of ignorance which now prevails over the whole of Hindostan."

